# A patient survey on outpatient physiotherapy services in Nepal: service received, and patients’ recommendations

**DOI:** 10.1186/s12913-025-13055-3

**Published:** 2025-07-01

**Authors:** Nishchal Ratna Shakya, Nistha Shrestha, Gillian Webb, Hellen Myezwa, Biraj Man Karmacharya, Ann-Katrin Stensdotter

**Affiliations:** 1 Department of Neuromedicine and Movement Science, Faculty of Medicine and Health Sciences, Norwegian University of Science and Technology (NTNU), Trondheim, 7491 Norway; 2 Department of Physiotherapy, Kathmandu University School of Medical Sciences, Dhulikhel, Kavre, Nepal; 3 Department of health services, Ministry of Health and Population, Epidemiology and Disease control division, Government of Nepal, Kathmandu, Nepal; 4 Faculty of Medicine, Dentistry and Health Sciences, The University of Melbourne, Melbourne, VIC Australia; 5 School of Therapeutic Sciences, University of the Witwatersrand, Johannesburg-Braamfontein, Gauteng, South Africa; 6 Department of Public Health, Kathmandu University School of Medical Sciences, Dhulikhel, Kavre, Nepal

**Keywords:** Service provision, Utilisation, Demand, Healthcare access, Nepal

## Abstract

**Background:**

A global rising burden of non-communicable diseases and disability increases the need of and demand on physiotherapy. In Nepal, knowledge is lacking about provision and utilisation of physiotherapy. This study aimed to explore patients’ perception about physiotherapy and the service received at their outpatient physiotherapy clinic. The rationale was to understand the needs and identify areas of potential improvement in service delivery.

**Methods:**

A cross-sectional survey with closed and open-ended questions was performed in the Bagmati province of Nepal at 29 health-centres of 6 districts including 20 patients per facility (*n* = 580). Descriptive and regression analysis were performed for closed ended questions and thematic content analysis used for the open-ended questions.

**Results:**

Among patients receiving physiotherapy, 55.3% were females and 48.1% belonged to the upper caste or advantaged ethnic groups, 76.4% were referred by doctors and 76.6% were paying out of pocket. Musculoskeletal (80.7%) and neurological (29.1%) conditions were most common with some comorbidity. The most frequent interventions were electrotherapy and resistance training (≈ 65%). Accessibility was explained by educational level, urban / rural area and travel time. Affordability was explained by age, mode of payment and treatment duration. Age, educational level, and mode of payment likewise explained satisfaction. Patients’ recommendations for improving physiotherapy were clustered on nine: satisfaction, availability, equipment, awareness, service improvement, human resources, affordability, adequate space and proper counselling.

**Conclusion:**

Based on the findings, Nepal should target political priorities including infrastructure, quality system for evidenced practice and clinical conduct, rural development and availability for the poor.

**Supplementary Information:**

The online version contains supplementary material available at 10.1186/s12913-025-13055-3.

## Introduction

Nepal is a low-income country with a high burden of disease and lack of effective healthcare systems. Similarly to the global trend, there is also in Nepal a shift from infectious toward non-communicable diseases due to changes in lifestyle and an ageing population accompanied by an increase of injuries with rising number of road traffic accidents and occupational hazards [1–3]. Physiotherapy as an integral part of the Nepal healthcare system in general and primary healthcare in particular could play a crucial role in addressing these health issues by health promotion, disease prevention and rehabilitation [4, 5]. Physiotherapy outpatient departments (OPD) generally receive patients with musculoskeletal, neurological or cardiorespiratory conditions but also many other disorders [6, 7]. Physiotherapy would thus serve as a critical source of service to tackle the disease burden and management of chronic conditions enhancing the efficiency of the healthcare system [8]. In Nepal, however, its role in the healthcare systems appears poorly understood and seems inadequately managed.

An effective healthcare system relates to providing equitable access for the entire population to affordable, high-quality healthcare including treatment, health promotion, prevention and rehabilitation services [9]. This aligns with the strategic plans of World Physiotherapy by 2026 recommending collaboration with international agencies to achieve affordable and equitable access to physiotherapy services [10]. Unfortunately, many countries, mainly lower- and middle-income, fail to meet adequate standards. Availability of physiotherapy services in rural areas, in contrast to urban settings, face multiple challenges affecting delivery of equitable physiotherapy services [11].

In Nepal, there are several potentially limiting factors for development, expansion, and access to physiotherapy services such as financial shortage, geographical and infrastructural challenges, as well as social and cultural considerations [12]. The country has a severe shortage of healthcare professionals, as was evident during COVID pandemic [13], physiotherapists being no exception.

Despite this, the number of health facilities, including physiotherapy service, are increasing. Still, inequity in access to healthcare in Nepal remains significant [14]. People with disability and suffering different health conditions often face broader social and economic implications [15]. Although physiotherapy is earning increased attention, patients’ access, needs, expectations, and satisfaction with what they receive remains unclear. There are presently no existing research platforms or registries available for statistics in Nepal to explore even the most basic and fundamental questions about healthcare provision [16].

Therefore, this study aimed to explore patients’ perception of physiotherapy and the services they received at their outpatient physiotherapy clinic to help understand the needs and identify areas of potential improvement in service delivery. The findings may contribute to guide policymakers and healthcare providers in Nepal in designing more effective and high-quality physiotherapy services aligned with recommendations and patients’ needs to improve the overall health and wellbeing.

## Methods

### Design and setting

**Fig. 1:**
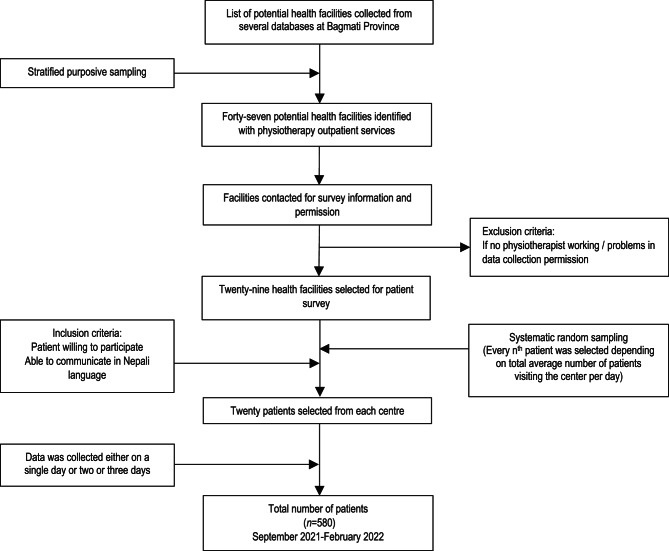
Recruitment procedure of patients

A cross-sectional patient survey with open and closed-ended questions, investigating experience and satisfaction with accessing and receiving physiotherapy was performed in Nepal, Bagmati Province. This is the second-most populous region with cultural and ethnic diversity, housing about 1/5 of the 29 million population. The region composes both rural and urban settings including the capital Kathmandu and has the majority of physiotherapy workforce personnel and services [17]. The districts and health facilities were selected using stratified purposive sampling aimed at maximum variation [18–20] performed in parallel with a previous study [21]. Six districts (Kathmandu, Bhaktapur, Lalitpur, Kavrepalanchowk, Makwanpur and Dolakha) out of 13 were selected to secure a mix of urban-rural settings with geographical and cultural diversity. Facilities were categorised into tertiary, secondary and medical college hospitals, and rehabilitation and physiotherapy clinics with a selection from a mix of metropolitan, urban-suburban and rural districts. Potentially eligible healthcare centres with physiotherapy OPD providing services for musculoskeletal, neurological, cardiorespiratory, paediatric and other disorders were identified. Primary contacts for the physiotherapy department in the facility were approached and information was provided by e-mail. Out of 47 potential centres contacted, eleven did not have operational physiotherapy, six had issues with data collection process and one did not respond. Finally, 29 of the health facilities across the six different districts agreed to participate (Table 1, Fig. 1).

**Table 1:** Selection of patients from health facilities

**Districts**	**Total centres**	**Tertiary hospital**	**Secondary hospital**	**Medical college **	**Rehabilitation/PT clinics**
Kathmandu^a^	12	5	3	2	2
Lalitpur^b^	5		1		4
Bhaktapur^b^	2		2		
Kavrepalanchok^b^	1			1	
Dolakha^c^	4		4		
Makwanpur^b^	5		3		2
Facilities total (n)	29	5	13	3	8
Patients total (n)	580	100	260	60	160

*PT* Physiotherapy

^a^Metropolitan

^b^Urban, sub-urban

^c^Rural district

### Participants

A systematic random sampling method was used [22] to select the patients in each facility, that is, selection based on a system of intervals in a numbered population, adapted to the estimated number of patients per day at a given site. Each site was asked to provide 20 participants (total number *n* = 580, Fig. 1). A contact person assigned the n^th^ patients from a registry for the day of the field visit. The onsite authority at the centre was informed whether an interested patient would be enrolled in the survey. Participants were eligible if they were receiving physiotherapy intervention, understood Nepali language and were willing to participate in the survey. For patients under the age of 16, their guardian was considered on their behalf for survey participation.

Ethical approvals were granted by the Nepal Health Research Council (NHRC-ERB Protocol No. 455/19), the Institutional Review Committee of Kathmandu University School of Medical Sciences / Dhulikhel Hospital (IRC-KUSMS 281/19), and the Norwegian Centre for Research Data (NSD 383963). Informed written consent was received from each participant or guardian prior to enrolment. Survey responses were anonymous, and without person identifiable data.

### Data collection

The survey contained both open and close-ended questions and was designed based on previous studies [23–25] and consultations with senior physiotherapists, two having working experience from physiotherapy OPDs in Nepal, two with academic and research backgrounds, and one government employed public health expert, to assure relevance and width of content. A draft survey was piloted at 5 different centres with 10 patients in each to ensure face validity, readability, and structure [26]. Feedback were incorporated and the final questionnaire was edited accordingly. The questionnaire contained three sections: categoric socio-demographic profile; Likert scale rating physiotherapy services; and open-ended recommendations on improving physiotherapy services in Nepal (Supplementary file*).* Data were collected by a research assistant, having no prior relationship with the participants or with their treating physiotherapist, and trained in collecting survey data using KoBoToolbox (https://www.kobotoolbox.org/). Each questionnaire took 7–10 min to complete. Data collection occurred from September 2021 to February 2022.

### Data analysis

For a confidence level of 95%, we found that a total of 580 participants represented approximately 20% of the number of patients in a month and the margin of error would lie within ± 3.26% of the surveyed value. Survey data collected with KoBoToolbox were extracted to Microsoft Excel spreadsheets. Quantitative data were processed in IBM SPSS version 28. Frequency analyses were adjusted for age: for marital stage > 19 years, for occupation and education > 18 years. Income-level for under-age patients (< 16 years of age), income was assumed as parents’, thus not adjusted. Normal distribution of data was assessed by QQ-plots. Associations between independent and dependent variables were analysed with Spearman’s rho. Then, stepwise linear regression analysis was used to predict which correlated background factors that could explain accessibility, affordability and satisfaction with physiotherapy. A model for each one of these three dependent variables was constructed. Collinearity was controlled for the independent variables.

Open ended responses were analysed with thematic content analysis [27] using a coding system [28]. First, the narrative data were analysed [29] and preliminary data coding was performed using a combination of deductive and inductive codes. Relevant texts were clustered into potential sub-categories and categories, which were reviewed, discussed and agreed upon [30]. In the last stage of analysis, an interpretative theme was conceptualised from the underlying content that could be identified across the main categories. MS Excel was used to facilitate sorting of narratives and quotations. The responses were translated from Nepali to English by the research assistant, coded and categorised by the first author. Soundness of the qualitative analysis was assured in accordance with the consolidated criteria for reporting qualitative research (COREQ) [31].

## Results

### Characteristics of patients

Table 2 presents socio-demographic characteristics of the patients. Patients spanned all age groups, but predominantly ranged between 30 and 59 years of age (55.1%). A slightly higher number were females (*n* = 321, 55.3%) and nearly half (*n* = 279, 48.1%) belonged to upper caste and advantaged ethnic groups. Most had secondary education (*n* = 177, 34.6%) while 117 (22.9%) had no formal education. Nearly all were married (*n* = 423, 83.8%), many were homemakers (*n* = 210, 41%), more than half lacked monthly income (*n* = 365, 62.9%) while an earning ranged from 2000 (*n* = 1, 0.2%) up to as high as 5,00,000 Nepalese rupees (*n* = 3, 0.5%) with mean income 54,858 per month (ca. 416 US dollars). Most respondents (*n* = 500, 86.2%) were from urban areas, whereas the remaining (*n* = 80, 13.8%) were from rural areas.

**Table 2:** Sociodemographic characteristics of participants

**Demographics**	**n (%)**
Age (means and SD)	41.8 (19.0)
<10	46 (7.9)
10-19	29 (5)
20-29	73 (12.6)
30-39	107 (18.4)
40-49	101 (17.4)
50-59	112 (19.3)
60-69	77 (13.3)
70-79	32 (5.5)
80-89	3 (0.5)
Gender	
Male	259 (44.7)
Female	321 (55.3)
Ethnicity	
Dalit	31 (5.3)
Disadvantaged janjati	67 (11.6)
Disadvantaged non-dalit terai caste	30 (5.2)
Religious minorities	4 (0.7)
Relatively advantaged janjati	169 (29.1)
Upper caste	279 (48.1)
Education (age=>19 years, *n*=512)^a^	
No formal education	117 (22.9)
Primary (1-8 grade)	85 (16.6)
Secondary (9-12 grade)	177 (34.6)
Bachelor	84 (16.4)
Master and above	49 (9.6)
Marital Status (age=>20 years,* n*=505)^b^	
Unmarried	63 (12.5)
Married	423 (83.8)
Divorced	3 (0.6)
Separated	16 (3.2)
Occupation (age=>19 years, *n*=512)^c^	
Homemaker	210 (41)
Agriculture	34 (6.6)
Business	43 (8.4)
Private	104 (20.3)
Government job	62 (12.1)
Student	30 (5.9)
Other job	35 (6.8)
Monthly income range (1 rupee ≈ 0.0076 US dollars)	
No income	365 (62.9)
1000-20000	48 (8.3)
21000-40000	91 (15.7)
41000-60000	45 (7.8)
61000-100000	15 (2.6)
101000-500000	16 (2.8)
Monthly income (n=215) range: 2000- 500000, means, [SD]	54858 [78352]^g^
Setting	
Urban	500 (86.2)
Rural	80 (13.8)
Patients from different districts	
Kathmandu^d^	239 (41.2)
Lalitpur^e^	80 (13.8)
Bhaktapur^e^	61 (10.5)
Kavrepalanchowk^e^	20 (3.4)
Dolakha^f^	80 (13.8)
Makwanpur^e^	100 (17.2)

Total number of respondents *n*=580, unless noted adjusted for age

^a^Secondary education is expected to achieve above 18 years of age

^b^Marriage is considered legal in Nepal above 19 years of age

^c^Respondents are expected to be engaged in some form of occupation above 18 years of age

^d^Metropolitan

^e^urban, sub-urban

^f^Rural district

^g^only respondents paid > 2000 answered this question

(Skewness 4, SE.166, kurtosis 18, SE.033)

### Patients’ use of and experience with physiotherapy outpatient services

Results of the closed-ended questions of the survey are shown in Tables 3, 4 and 5. The majority of the patients (*n* = 443, 76.4%) were referred by doctors. Most had musculoskeletal related problems (*n* = 468, 80.7%) followed by neurological problems (*n* = 169, 29.1%). Half had chronic problems (*n* = 294, 50.6%) and some had several diagnoses. The treatments received were predominantly electrotherapy (*n* = 392, 67.6%) followed by resistance training (*n* = 364, 62.8%), endurance training (*n* = 339, 58.4%) and home programs (*n* = 280, 48.3%). Some received more than one type of intervention. Most, 431 (74.3%) were interviewed during their follow up session. Totally 515 (88.8%) patients had follow-up plans where 7 sessions were most common (*n* = 92, 15.9%). Follow up until recovery was reported by 68 (11.7%) whereas 65 (11.2%) patients had no planned follow up session. Most patients (*n* = 367, 63.3%) had a treatment duration for less than one month. Very few patients (*n* = 22, 3.8%) were referred to other departments or centres, mostly for radiologic examination (MRI, CT, or X-ray), prosthetics and orthotics. The most common means of transportation to reach the facility was by public bus (*n* = 217, 37.4%). Among all the respondents, 444 (76.6%) patients reported that they paid for the treatment out of pocket. A large proportion (*n* = 520, 89.7%) reported that service was easy or very easily accessible and several (*n* = 482, 83.1%) reported that the service was affordable or very affordable. Many (*n* = 573, 98.8%) of the respondents were satisfied or very satisfied with the services. Some gave reasons for satisfaction in the open-ended questions. Others, even though satisfied, had several suggestions for improvement.

**Table 3:** Contextual factors for physiotherapyservices in Bagmati Province Nepal as reported by patients

**Independent variables**	**N (%)**
Referral	
Doctor	443 (76.4)
Self-referral	73 (12.6)
Family/Friends	53 (9.1)
Advertisement	10 (1.7)
Others	1 (0.2)
Patients with problems	
Musculoskeletal	468 (80.7)
Neurological	169 (29.1)
Pain	36 (6.2)
Spinal	26 (4.5)
Trauma	27 (4.7)
Cardiovascular	4 (0.7)
Respiratory	1 (0.2)
Others	41 (7.1)
Condition	
Acute	85 (14.7)
Sub-acute	201 (34.7)
Chronic	294 (50.6)
Physiotherapy interventions	
Electrotherapy	392 (67.6)
Motor relearning	78 (13.4)
Endurance training	339 (58.4)
Resistance training	364 (62.8)
Flexibility	221 (38.1)
Balance	75 (12.9)
Supervised training in clinic	47 (8.1)
Home Program	280 (48.3)
Massage	12 (2.1)
Consultation	55 (9.5)
Any other treatments	40 (6.9)
Patient treatment session	
First session	149 (25.7)
Not the first session	431 (74.3)
Follow up plans	
No	65 (11.2)
Yes	515 (88.8)
Follow up sessions	
1 Day	19 (3.3)
2 Day	34 (5.9)
3 Day	30 (5.2)
4 Day	35 (6)
5 Day	53 (9.1)
6 Day	21 (3.6)
7 Day	92 (15.9)
Until recovery	68 (11.7)
Treatment period	
<1 month	367 (63.3)
1-3 months	148 (25.5)
4-6 months	16 (2.8)
7-12 months	7 (1.2)
>12 months	42 (7.2)
Referral from physiotherapy to other departments	
No	558 (96.2)
Yes	22 (3.8)

Total number of respondents *n* = 580

Note that number of total responses may exceed 580 as one patient can have multiple resposes

Percentage is calculated out of total number of respondents (*n =580*) irrespective of multiple responses per person

**Table 4:** External constraints for accessibility and affordability of physiotherapy services as reported by patients

**Independent variables**	**N (%)**
Mode of transportation (*N*=643)	
Walk	140 (24.1)
Motorcycle	199 (34.3)
Private car	53 (9.1)
Local bus	217 (37.4)
Taxi	27 (4.7)
Other	7 (1.2)
Mode of payment	
Out of pocket	444 (76.6)
Insurance coverage	94 (16.2)
Other	48 (8.3)
Insurance coverage	
Government	92 (15.86)
Private	2 (0.34)
Time to reach the centre	
Less than 1 hour	495 (85.3)
1-3 hours	81 (14)
Half day (1/2 day)	3 (0.5)
One day (1 day)	1 (0.2)

Total number of respondents *n* = 580

**Table 5:** Perception of physiotherapy services in Bagmati Province, Nepal as reported by patients

**Independent variables**	**N (%)**
Accessibility for service	
Very difficult	11 (1.9)
Difficult	36 (6.2)
Neither difficult nor easy	13 (2.2)
Easy	149 (25.7)
Very easy	371 (64.0)
Service affordability	
Very affordable	271 (46.7)
Affordable	211 (36.4)
Neither affordable nor expensive	57 (9.8)
Expensive	32 (5.5)
Very expensive	9 (1.6)
Satisfaction of patients	
Not satisfied	1 (0.2)
Not sure	6 (1.0)
Satisfied	191 (32.9)
Very satisfied	382 (65.9)

Total number of respondents *n* = 580

### Associations between background variables with accessibility, affordability and satisfaction

Accessibility was better for patients living in urban areas (*r* =.486), having higher education (*r* =.203), and chronic condition (*r* =.119), and worse with longer travelling time (*r* = −.447) and paying out of pocket (*r* = −.108). Younger patients (*r* = −.185) and those paying out of pocket (= − 0.357) found the services less affordable. Patients in the urban area, those with chronic condition and longer treatment duration found the services more affordable (*r* =.113, 0.132 and 0.120 respectively). Satisfaction was lower for younger patients (r=−.116) and those paying out of pocket (=−.137), while higher education level and higher income was associated with higher satisfaction (*r* =.155 and 0.115) (Table 6).

**Table 6:** Descriptive statistics and correlation coefficient for dependent outcome variables

**Variables**	**Accessibility**			**Affordability**			**Satisfaction**		
	r	95% CI		r	95% CI		r	95% CI	
Age group	.011	-.073	.095	-.185**	**-.264**	**-.103**	-.116**	**-.198**	**-.033**
Gender	-.024	-.108	.060	-.035	-.119	.049	-.058	-.142	.026
Ethnicity	.040	-.044	.123	.006	-.078	.089	.039	-.045	.123
Rural-Urban	.486**	**.419**	**.547**	.113**	**.030**	**.195**	.018	-.066	.101
Education^a^	.203**	**.121**	**.282**	.026	-.058	.110	.155**	**.073**	**.236**
Income	.038	-.046	.121	.027	-.057	.110	.115**	**.032**	**.197**
Acute-Chronic	.119**	**.035**	**.201**	.132**	**.048**	**.213**	.044	-.040	.128
Treatment duration	.045	-.039	.128	.120**	**.037**	**.202**	.031	-.052	.115
Mode of payment	-.108**	**-.191**	**-.024**	**-.357** **	**-.429**	**-.282**	-.137**	-.219	-.054
Time to travel	-.447**	**-.512**	**-.377**	-.001	-.085	.083	.027	-.056	.111

^a^Education: age < 19 years were removed

**p < 0.01 (2-tailed); *p < 0.05; *N*=580

### Explanatory power of correlated background variables with accessibility, affordability and satisfaction

The dependent variables were explained by both positively and negatively correlated independent variables. Urban living and higher education had a positive effect on accessibility, while longer travel time had negative effect (*R*^2^ = 0.474, *p* <. 001). Younger age and payment out of pocket had a negative effect on affordability, while shorter treatment period had a positive effect (*R*^2^ = 0.188, *p* <.001). Higher education had a positive effect on satisfaction while younger age and paying out of pocket had a negative effect, *R*^2^ = 0.043, *p* <.001 (Table 7).

**Table 7:** Stepwise regression analysis showed how grouped independent variables explained dependent variables for physiotherapy

**Dependent variables**	**Independent variables**		**β**	***p*** **-value**	**CI 95%**	
Accessibility	Demographics	Rural/Urban	.391	<.001	0.892	1.246
		Education	.102	.001	0.031	0.122
		Ethnicity	.045	.142		
		Acute-Chronic	-.015	.635		
	Other variables	Time to travel	-.407	<.001	-1.140	-0.828
		Mode of payment	-.033	.305		
Affordability	Demographics	Age group	-.214	<.001	-0.143	-0.068
		Acute-Chronic	.028	.500		
		Rural-urban	-.024	.566		
	Other variables	Mode of payment	-.291	<.001	-0.574	-0.341
		Treatment duration	.174	<.001	0.083	0.214
Satisfaction	Demographics	Education	.131	.002	0.020	0.086
		Age group	-.107	.009	-0.050	-0.007
		Income	.066	.078		
	Other variables	Mode of payment	-.095	.021	-.151	-.012

95% CI (confidence interval): values for excluded factors in the model (*p*>.05) are not included

### Patients’ comments and advice with regard to received physiotherapy services

**Fig. 2:**
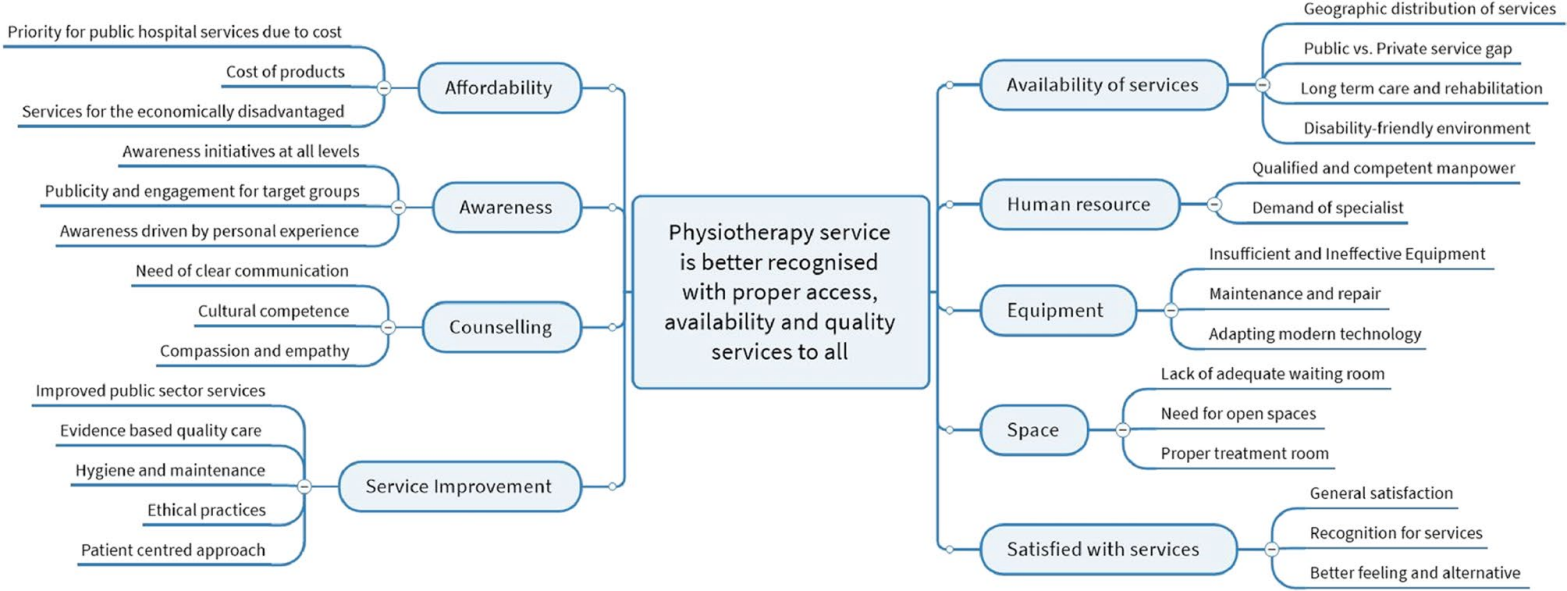
The identified sub-categories grouping into categories and conceptualised into one main theme summing up open-ended survey responses on received physiotherapy service from 375 patients

All 580 filled out the open-ended part of the survey, while 375 gave comments or advice. The remaining 205 did not respond or had no opinion giving responses such as: “*I am new to the service”; “I do not know much about it”; I don’t know what to say”; “This is my first day of treatment*,* so can’t tell anything”*, or *“this is not my field”.* From the 375 comments and advice, a number of sub-categories were sorted into nine main categories that were conceptualised into an overarching theme as: *“Physiotherapy service is better recognised with proper access*,* availability and quality services to all”* (Fig. 2). The majority of responders expressed satisfaction with the received service, but many had at the same time comments on the received service and also gave advice.

*Satisfaction* with the received service was expressed by 39.5% of the respondents as exemplified from the following citations from the open-ended questions:

• *“I don’t have any complaints*,* services provided are good with reasonable cost.”*
• *“Everything is good*,* physiotherapy works.”*
• “*Physiotherapy relieved my pain and is better than operation*.”
• *“In many hospitals*,* physiotherapist only give machine as treatment. Here*,* they provide the machine treatment as well as exercise. So*,* I find it very well.”*

*Availability of services* was commented by 20% and comprised critical issues and perspectives about physiotherapy services in Nepal, particularly concerning accessibility, equity, and infrastructure where some emphasised:

• *“Physiotherapy should not be concentrated just in Kathmandu but in other places as well*,* especially rural areas.”*
• “*The gap in physiotherapy services available between government and private hospitals should be reduced*….”
• *“Patient with long term care needs to stay in a hospital. We need to have patient admission system for rehabilitation services.”*
• *“There is a (X) government hospital*,* but it does not have child friendly environment. There is one non-government organisation*,* but it is far.”*

*Equipment* was commented by 14.7% who generally found it insufficient and ineffective. Many centres lacked adequate physiotherapy equipment, and the existing equipment were often ineffective or not functioning properly. The patients remarked that:

• *“They should also repair their machines frequently.”*
• *“The centre should get advanced machines to replace the old ones.”*

*Awareness* was driven both by personal experience and opinions, expressed by 11.7% targeting importance of physiotherapy and its benefits:

• *“There should be awareness from school level*,* camps should be conducted…”*
• *“As a national player*,* I would say if seminars were conducted from this organisation*, *it would be more helpful..”*
• *“I came to know about physio because I had pain and needed treatment.”*

*Improvement in physiotherapy services* was pointed out by 10.9% from many different perspectives from government priorities, modes of intervention and evidence-based treatment. Complaints were about hygiene, poor time management and lack of consultation. It was an opinion that profit rather that provision seemed to be the objective as expressed by some patients:

• *“Services in government hospitals should be better than private to avoid expensive charges but there is no focus or priority from government.”*
• *“Focus should be more on exercise and not just machine.”*
• *“Pain relief is only during treatment sessions*,* so they should provide quality treatment based on research.”*
• *“They should change bed sheets and sanitise well time to time….”*
• *“…it is like how to get profit than service provision… no one to one consultation… there should be proper time management.”*

*Human resources* were viewed by 8.0% as a critical aspects of workforce development and diagnostic rigor in physiotherapy services. This was emphasized by a couple of patients expressing:

• *“There is a need of more qualified*,* trained*,* and skilled staff.”*
• *“Treatment should be done after proper diagnosis and problem identification….”*

*Affordability* was raised by 6.1% as a barrier to accessing physiotherapy. High costs in the private sector and expensive support devices were examples of what some could not afford:

• *“Services in government hospitals should also be good*,* so that we don’t have to come to private. We can’t afford prolonged treatment.”*
• *“Orthosis and prosthetic devices are very expensive”*.

Others pointed out the need for government intervention to ensure affordability and equity as:

• *“Poor people should get physiotherapy free of cost.”*
• *“Government should give some compensation. The private service is very expensive*,* common people cannot afford.”*

*Space* was highlighted by 5.3% as infrastructural and operational challenges in physiotherapy facilities and gave examples such as:

• *“There is a lack of sitting area for elderly patient and have to wait long due to the number of patients….”*
• *“There should be more open spaces in this department…”*
• *“Treatment rooms are very small*,* congested and there is no privacy…”*

*Counselling* was exclusively singled out by 4.0% for the importance of effective communication, patient-centred care and respectful interaction in physiotherapy services. Patients expressed that they [the therapist] should:

• *“…better explain the process and treatment…till when it must be done. explain about the importance”*.
• *“…listen to patient*,* understand the problem first and treat accordingly… Some staff don’t often communicate…”*
• “… should care and respect while treating their patient and should not be rude….”

## Discussion

The main result of this survey showed that 98.8% of the patients were satisfied with the physiotherapy they received, 83.1% found it affordable and 89.7% found it accessible. Most, 76.4%, were referred from a doctor and 80.7% had musculoskeletal problems. Electrotherapy and exercise were common interventions (≈ 65%), similar to a previous study [11]. Nearly 90% were on a follow-up plan, which is proven necessary for recovery of various conditions [32, 33]. Nearly 40% of the patients had constructive suggestions about improvements.

The results of the present study should be interpreted in the light of that most responders to the survey were from upper or advantaged castes (≈ 80%) and that more than half (≈ 60%) had secondary or higher education. This may explain the high affordability and accessibility as the majority if the responding patients can be considered prioritized. Even though approximately half of the respondents claimed no income, notably almost half were homemakers, and more than half were women. More than 80% were married. In Nepal, the most common family constellation is that the woman takes care of the home and is supported by the husband. It is not unusual that male family members work abroad with high income to support the family [34]. Women and marginalised groups are generally engaged mostly in the agricultural sector as force labour either with no formal income or low wage [35]. This also explains the very large discrepancies in income.

Notably, the government has recently launched a strategy and implementation plan for universal health coverage to ensure that the urban poor can receive treatment in accessible places [36]. A social health insurance program was also established already in 2015, aiming to provide financial protection and facilitate access to healthcare services for the poor, disadvantaged and marginalised populations. Regrettably, the public awareness and perception about the program appear inadequate and poor with weak adherence and high dropout rates from the program [37, 38]. Even after several years of operation, this health insurance program had not started in the Kathmandu and Lalitpur Metropolis according to the Nepal National insurance board 2023 report [39]. These can be the reasons that still most respondents in our study claimed payment out of pocket, implying that they were without any health insurance coverage, either government or private. This finding is similar to a recent health insurance enrolment review study published in 2024 that reported overall low coverage of government or any other type of health insurance as the largest problem [40].

Not surprisingly, cost and location proximity to health care services were indicators for perceived access to physiotherapy services, also shown elsewhere [41]. Thus, the perceptions of the respondents in the present survey were influenced by who they were and where they lived. Notably, 25 out of 29 centres were in urban or sub-urban areas, with an overrepresentation of Metropolitan Kathmandu. Despite an effort to recruit several centres from rural areas, we were generally unable to locate physiotherapy services at available healthcare facilities, either public or private. Even when identified, the service was generally non-operational due to a variety of reasons. This implied lack of availability of physiotherapy service making it difficult or impossible to collect data from rural facilities. This fact exposed a concerning gap in service provision between urban and rural areas in Nepal which is particularly problematic given the high prevalence of non-communicable and chronic diseases in these areas [42]. Amending this lack, hospitals or organisations provide outreach service in physiotherapy by field visits or organising camps to many rural health-service stations [43–45]. Data collected at such visits might have revealed different results but was not undertaken due to logistic reasons and the fact that data was collected during the Covid pandemic when many visits and camps were cancelled. The voices from people with lower caste and marginalised ethnic groups living in the very remote areas, also found in Bagmati Province, would however still have been left out as they are not likely to visit these outreaches due to topographic challenges with no roads and unequal access to healthcare, particularly rehabilitation services [46–48].

Despite these challenges, Nepal has in recent years made progress in expanding access to healthcare services. In the National health policy 2019, expansion and integration of physiotherapy services was planned at federal, provincial and local levels by also promoting private and non-governmental organisations to establish rehabilitative and palliative service centres [49]. The government launched Nepal Health Sector Strategic Plan 2015–2020 (updated 2022–2030), aiming to improve accessibility, quality, and affordability of health services, including physiotherapy [50]. The strategy prioritises upscaling of services in rural and remote areas, strengthening of health infrastructure, and development of human resources for health. The government has recently adopted a 10-year action plan highlighting the need for rehabilitation services in the country [51]. In light of the present findings, we emphasise that the concerned authorities should immediately act on properly implementing the regulations as stated in the Nepal government’s public health service regulations 2020 [52]. This regulation considers physiotherapy under specialised services to be further promoted and states that physiotherapy clinics and its services should be prioritised at general hospitals, general ayurveda hospitals, rehabilitation centres and geriatric care centres with more than 25 beds.

Of the patients responding to the survey, 80.7% reported having musculoskeletal disorders and 50.6% had chronic conditions corroborating a previous study from Nepal [53]. Chronicity may be prevented by early detection and intervention [54] at the primary healthcare level. The situation assessment of rehabilitation in Nepal 2022 has reported that there are no rehabilitation professionals in the government sector for primary healthcare. The new basic healthcare package is however adopting a task shifting approach for a few limited interventions which may help to integrate physiotherapy services at this level [17]. Our results show that 76.4% were referred to physiotherapy by their doctor. As we found that nearly one of four visits were not doctor referrals, this suggests the option of physiotherapy as first choice of consultation [55], which is positive in the context of health literacy. It is therefore recommended that referrals from medical doctors should be appreciated and encouraged and that awareness campaigns should advertise utilisation of physiotherapy services.

Of all identified treatments, electrotherapy was dominating (67.6%). This suggests that such passive interventions are often preferred as shown in previous studies [56]. Despite the lack of evidence whether electrotherapy has any physiological effect [57] or arguments against its use in contemporary practice [58] and lack of evidence of therapeutic effect [59], the patients’ perception of having received treatment may be of value to motivate adherence to physiotherapy intervention. Our results show that most patients did receive several modes of intervention where resistance and endurance training were next to most common (≈ 60%). The respondents were generally satisfied with the physiotherapy they received, but at the same time had many suggestions for improvement, consistent with previous findings from other studies [11, 60]. The narratives from the open-ended questions complemented the findings derived from the closed-ended questions. The respondents raised concerns mainly about service availability, equipment and awareness about physiotherapy. Specifically, it was highlighted that physiotherapy services should be available in different areas, also rural, and in all hospitals, prioritising public hospitals. Other suggestions concerned infrastructure and equipment. Some respondents found the equipment old, unreliable, and out of function and recommended maintenance of existing and investments in more and new equipment. They also commented on the need for spacious physiotherapy outpatient clinics with adequate sitting area and proper time management for patient treatment. This underlines the needs especially in neuro-rehabilitation, but also any other physiotherapy interventions, requiring adequate time, space and equipment [3]. The World Physiotherapy revised guidelines 2011 for standards of physiotherapy practice specifies physical settings with adequate space according to the number and types of patients, including reception and waiting areas with consideration to people with disabilities. It also recommends that equipment should be inspected and maintained routinely [61]. Our findings along with international guidelines emphasise that the concerned authorities need to conduct monitoring and evaluation of health facilities securing that minimum standards for hospitals in accord with the Ministry of Health and Population [62] are implemented effectively for strengthening the health services and management to ensure quality healthcare.

Further comments regarded communication and cultural competence. These are integral elements to facilitate provision of effective and appropriate physiotherapy services. Responders commented that physiotherapist should teach the patient home exercises properly, understand the patient’s problem and disorder first before initiating the treatment. The therapist should also treat the patient with care, empathy, equal respect and professional conduct. Absence of these elements in patient contact violates communication and cultural competence standards of practice by World Physiotherapy [61]. Patients’ comments agree with a previous survey reporting only 1% of the physiotherapy workforce in Nepal was involved in patient counselling [53]. This emphasises that Nepal Physiotherapy Association or a regulatory body such as the Nepal Health Professional Council need to develop core guidelines for Nepalese physiotherapist for such competencies.

### Strengths and limitations of the study

This is the first multi-centre patient survey in Nepal to explore patients experience with outpatient physiotherapy services. The intention of urban-rural inclusion of clinics and a broad representation of patients was to assure a comprehensive and representative picture of patients’ perception of physiotherapy service. This was limited by the low representation of responders from rural areas, due to unavailability of services, a finding in itself. We are also aware of possible inclusion bias as clinics volunteering for participation may have done so to promote themselves and by being allowed to include patients for the survey may have selected those with a positive attitude from prioritised social groups likely to give desirable responses.

## Conclusion

Based on this survey with a majority of rather well-situated responders from urban areas, high satisfaction with services perceived as both affordable and easily accessible, was found. Still, there were many suggestions for improvement that sometimes seemed to contrast the high satisfaction. A caveat was the low representation from rural Nepal and from marginalised groups. This is of concern and implies that Nepal needs to systematically integrate physiotherapy and rehabilitation services into the overall health policy framework and develop proper planning of human resources and infrastructure to meet the current and future demands. The recommendations based on our survey data provide feasible actions that may be taken at leadership and provider level. Recommendations to improve physiotherapy services in Nepal should target political priorities including infra structure, quality system for evidence-based practice and clinical conduct, and rural development and availability for the poor.

## Supplementary Information

Supplementary Material 1.

## Data Availability

The questionnaire used in this study are available in the supplementary file.
