# Supplementary material for: A patient survey on outpatient physiotherapy services in Nepal: service received, and patients’ recommendations
Source: BMC Health Serv Res. 2025 Jul 1;25:839. doi: 10.1186/s12913-025-13055-3 (PMC12211125; doi:10.1186/s12913-025-13055-3)
Supplement: Supplementary file 1 — Supplementary Material 1. [file 12913_2025_13055_MOESM1_ESM.docx]

***Appendix 1. Questions to the patients***

Do you wish to provide consent for this research? Yes/No

**Patient General Information**

1. Facility code:
2. Patient code:
3. Age:
4. Gender

- Male
- Female
- Prefer not to say

1. Ethnicity

*(Ask the caste of participants if unable to determine the ethnicity)*

- Dalit
- Disadvantaged janajati
- Disadvantaged non-dalit terai caste
- Religious minorities
- Relatively advantaged janajati
- Upper caste

1. Education

- No formal education
- Primary (1-8 grade)
- Secondary (9-12 grade)
- Higher (Bachelor)
- Highest (Master and above)

1. Marital status

- Unmarried
- Single
- Married
- Divorced
- Separated
- Widow/widower

1. Occupation

- Home maker
- Agriculture
- Business
- Private job
- Government job
- Student
- Others, mention:

1. Monthly income

- Yes
- No

*If yes, how much do you earn in a month in average?*

1. From which province, have you visited here for your treatment?

- Province 1
- Province 2
- Province 3
- Province 4
- Province 5
- Province 6
- Province 7

**Physiotherapy Related Information**

1. Referral

- Doctor
- Self-referral
- Family/friends
- Advertisement
- Others, mention:

1. What is your problem related to:

- Musculoskeletal
- Neurological
- Pain
- Spinal
- Trauma
- Cardiovascular
- Respiratory
- Others, mention:

1. Since when you had a problem?

- Acute (Immediate/Last for few days)
- Sub-acute (<3months)
- Chronic (Long term:>3-6 months)

1. Treatment/Intervention

- Electrotherapy
- Motor re-learning
- Endurance training
- Resistance training
- Flexibility
- Balance
- Supervised training in clinic
- Home Program
- Massage
- Consultation
- Other treatments, mention if others:

1. Treatment session

- First session
- Not the first session

*Total number of treatment session if it is not the first session:*

1. Any follow up sessions planned?

- Yes
- No

*Mention the follow-up session planned (if any):*

1. Since when are you receiving the treatment?

- <1 month
- 1-3 months
- 4-6 months
- 7-12 months
- >12 months

1. How do pay for your treatment cost?

- Out of pocket
- Insurance coverage
- Others, *mention:*

*If insurance coverage:*

- Government
- Private
- Both

1. Are the services affordable?

- Very affordable
- Affordable
- Neither affordable nor expensive
- Expensive
- Very Expensive

1. Mode of transport

- Walk
- Motorcycle
- Private car
- Local bus
- Taxi
- Other, *mention:*

1. Time to travel:

- <1 hour
- 1-3 hours
- Half day (1/2 day)
- One day (1 day)
- >1 day

1. Accessibility to the facility:

- Very difficult
- Difficult
- Neither difficult nor easy
- Easy
- Very easy

1. Have you been referred from physiotherapy department to another department/health professional for further diagnosis or treatment?

- Yes
- No

*If yes, mention for what?*

1. Overall, are you satisfied with the physiotherapy services?

- Not satisfied
- Not sure
- Satisfied
- Very satisfied

**Open ended survey question:**

1. Do you have any recommendations how physiotherapy service can be better in Nepal?
